# Ammonium-Dependent Shortening of CLS in Yeast Cells Starved for Essential Amino Acids Is Determined by the Specific Amino Acid Deprived, through Different Signaling Pathways

**DOI:** 10.1155/2013/161986

**Published:** 2013-08-26

**Authors:** Júlia Santos, Cecília Leão, Maria João Sousa

**Affiliations:** ^1^Life and Health Sciences Research Institute (ICVS), School of Health Sciences, University of Minho, 4710-057 Braga, Portugal; ^2^ICVS/3B's-PT Government Associate Laboratory, Braga/Guimarães, Portugal; ^3^Molecular and Environmental Biology Centre (CBMA), Department of Biology, University of Minho, 4710-057 Braga, Portugal

## Abstract

Ammonium (NH_4_^+^) leads to chronological life span (CLS) shortening in *Saccharomyces cerevisiae* BY4742 cells, particularly evident in cells starved for auxotrophy-complementing amino acids (leucine, lysine, and histidine) simultaneously. Here, we report that the effect of NH_4_^+^ on aging yeast depends on the specific amino acid they are deprived of. Compared with no amino acid starvation, starvation for leucine alone or in combination with histidine resulted in the most pronounced NH_4_^+^-induced CLS shortening, whereas starvation for lysine, alone or in combination with histidine resulted in the least sensitivity to NH_4_^+^. We also show that NH_4_^+^-induced CLS shortening is mainly mediated by Tor1p in cells starved for leucine or histidine but by Ras2p in cells starved for lysine, and in nonstarved cells. Sch9p protected cells from the effect of NH_4_^+^ under all conditions tested (starved or nonstarved cells), which was associated with Sch9p-dependent Hog1p phosphorylation. Our data show that NH_4_^+^ toxicity can be modulated through manipulation of the specific essential amino acid supplied to cells and of the conserved Ras2p, Tor1p, and Sch9p regulators, thus providing new clues to the development of environmental interventions for CLS extension and to the identification of new therapeutic targets for diseases associated with hyperammonemia.

## 1. Introduction

In all living organisms, cell survival is mediated by metabolic regulation in response to environmental conditions. This regulation is conserved from yeasts to mammals and is mediated by complex nutrient signaling pathways that control the necessary metabolic changes that take place when environmental conditions change [1]. In yeast, when nutrients are depleted, cells undergo a growth arrest phase characterized by downregulation of growth signaling pathways and upregulation of several processes, such as accumulation of carbohydrates, autophagy, and stress resistance [2, 3]. The length of time these nondividing yeast cells remain viable for is defined as the chronological life span (CLS) of the population [4]. The composition of the culture medium can modulate CLS, and therefore, culturing cells in different media leads to differences in CLS [5]. Manipulation of several single components of the culture medium is known to extend CLS, such as reducing glucose concentration (known as caloric restriction-CR) or manipulating the supply of amino acids [5–9]. Several studies in the literature report different effects of amino acids on life span regulation, depending on which amino acid is deprived [6–10]. In this context, it is known that starvation for nonessential amino acids (strains without auxotrophies) used as preferred nitrogen sources can extend CLS [11–13], while starvation for auxotrophy-complementing amino acids (essential amino acids) reduces CLS [7, 10]. However, not all essential amino acids contribute equally to the effects on CLS. In fact, it has been described that leucine plays a more important role in CLS extension in auxotrophic strains [6, 10] and that extra supplementation of leucine promotes CLS extension in standard 2% glucose medium [6]. Recently, it has also been shown that leucine influences autophagy and extension of CLS during CR [14].

The target of rapamycin complex 1 (TORC1) controls cell growth in response to the availability of nutrients, including amino acids [15, 16]. The TOR pathway responds to nitrogen by regulating processes such as the transcription of genes involved in nitrogen metabolism: nitrogen catabolite repression (NCR) sensitive genes, amino acid biosynthesis genes (general amino acid control pathway-GAAC), retrograde response genes (RTG-Pathway), and genes involved in the stability of amino acid permeases and autophagy [17, 18]. In mammalian cells, amino acids, predominantly leucine, regulate mTOR by controlling the ability of the positive regulator Rheb-GTP to activate mTORC1. The fundamental role of leucine in TORC1 regulation has been demonstrated through the observation that withdrawal of leucine alone is almost as effective in downregulating TORC1 as withdrawal of all amino acids combined [19]. In yeast, the EGO complex is an upstream regulator of TORC1, thought to be responsible for amino acid signaling to TORC1. During leucine starvation, TORC1 activation by this complex is disrupted, which results in a reduction in Sch9p phosphorylation and slow growth [20, 21].

The protein kinase A (PKA) pathway is involved in the regulation of metabolism, stress response, and proliferation, responding to the presence of a rapidly fermentable sugar and other essential nutrients sustaining growth, such as amino acids and phosphate [16, 22]. Readdition of nitrogen (amino acids or ammonium) to cells starved for nitrogen activates the PKA pathway through plasma membrane sensors known as transceptors. Sch9p is a protein kinase that shares many targets with PKA and TORC1, and different interactions between these pathways, either cooperating or antagonizing their effects, have been described [23]. It was shown that Sch9p mediates PKA activation in the fermentable growth medium induced (FGM) pathway, in response to amino acid and ammonium, but not in phosphate-induced activation [24].

We have previously shown that decreasing the ammonium (NH_4_^+^) concentration in the culture medium extends the CLS of *Saccharomyces cerevisiae* BY4742 cells [25]. NH_4_^+^ reduced the CLS of cells cultured to stationary phase under both standard amino acid supplementation and amino acid restriction conditions in a concentration-dependent manner, and a significant increase in cell survival was observed when the starting NH_4_^+^ concentration in the medium was decreased. We also showed that when stationary phase cells were transferred to water, the CLS was also significantly shortened by addition of NH_4_^+^, indicating that NH_4_^+^ alone could induce the loss of cell viability observed in culture media. The negative effects of NH_4_^+^ were particularly evident in cells cultured or incubated under restriction of auxotrophy-complementing amino acid markers (leucine, lysine, and histidine). These negative effects of NH_4_^+^ do not appear to require its metabolization. The PKA and TOR pathways were involved in NH_4_^+^-induced CLS shortening, but deleting* SCH9* did not revert the decrease in cell viability despite abolishing PKA activation in response to NH_4_^+^, suggesting Sch9p plays an independent role in cell survival [25].

Here, we show that NH_4_^+^ toxicity during yeast aging in water depends on the specific starved auxotrophy-complementing amino acid. Sch9p, contrary to Tor1p and Ras2p, mediates cell survival in response to NH_4_^+^ in all starvation conditions through the phosphorylation of Hog1p. Our results provide new insights in the modulation of CLS by NH_4_^+^, linking NH_4_^+^ toxicity to amino acid limitation. This scenario of enhanced NH_4_^+^ toxicity in amino acid starvation conditions is present in hyperammonemic patients, who are often on dietary protein restriction [26]. The use of a simpler model like yeast can help elucidate the underlying mechanism involved in the modulation of conserved signaling pathways, in response to NH_4_^+^.

## 2. Materials and Methods

### 2.1. Strains and Growth Conditions

*Saccharomyces cerevisiae *strain BY4742 (*MAT*a *his3Δ*1 *leu2Δ*0 *lys2Δ*0 *ura3Δ*0) (EUROSCARF, Frankfurt, Germany) and the respective knockouts in *HOG1*, *RAS2*, *SCH9,* and *TOR1 *genes were used. For experiments with nonstarved and amino-acid-starved cells, the strains were first cultured at 26°C, 150 rpm, in defined minimal medium (SC medium) containing 0.17% yeast nitrogen base without amino acids and without ammonium sulphate (Difco, BD), supplemented with 0.5% (NH_4_)_2_SO_4_, with appropriate amino acids and nucleotide base (50 mg/L histidine, 50 mg/L lysine, 300 mg/L leucine, and 100 mg/L uracil) and 2% D-glucose, to exponential phase (OD_600_ = 1.0–1.5). These cells were harvested and resuspended in (A) SC medium containing 4% glucose (nonstarved cells) or in (B) SC medium containing 4% glucose and lacking (1) amino acids (aa-starved cells); (2) leucine (Leu-starved cells); (3) histidine (His-starved cells); (4) lysine (Lys-starved cells); (5) histidine and lysine (His-Lys-starved cells); (6) leucine and lysine (Leu-Lys-starved cells), and (7) leucine and histidine (Leu-His-starved cells). After 24 hours, cells were collected by centrifugation, washed three times with water, and resuspended at a cell density of about 3.8 × 10^7^cells/mL in water (pH 7.0), or water with (NH_4_)_2_SO_4_ (0.5%, pH 7.0). Viability of 24-hour-starved cultures was considered to be 100% of survival, and this was considered day 0 of the experiment. pH 7.0 was maintained throughout the experiment in cultures with adjusted pH. Cell viability of culture aliquots was assessed by CFU at day 0 (100% viability) and in subsequent days, as indicated. For CFU determination, diluted samples were incubated for 2 days at 30°C on YEPD agar plates. 

### 2.2. Trehalase Activity

Trehalase activity was determined according to [27]. Briefly, crude enzyme extracts were obtained by resuspending the cell pellet in ice-cold 50 mM MES/KOH buffer (pH 7.0) containing 50 *µ*M CaCl_2_ and adding a roughly equal volume of 0.5 mm diameter glass beads, followed by vigorous mixing during 1 minute intervals interspersed with periods of cooling on ice. The extracts were then dialyzed overnight at 4°C in a dialysis cellulose membrane (Cellu Sep H1, Orange). The dialyzed extract was then used to assess trehalase activity by measuring the released glucose using a glucose oxidase assay (GOD, Roche). Protein concentration was determined using the Bradford assay (Bio-Rad, Germany) according to the manufacturer's instructions.

### 2.3. Western Blot Analysis

Western blot analysis was performed according to [28]. Briefly, protein lysates were separated on 12.5% SDS-PAGE gels and transferred to polyvinylidene fluoride membranes (Hybond-P; Amersham). The membranes were blocked with 5% bovine serum albumin (BSA) in Tris-buffered saline (TBS, 50 mM Tris, 150 mM NaCl, and pH 7.6) containing 0.05% Tween 20 for 1 h at room temperature. Membranes were then incubated overnight at 4°C with primary antibodies directed against Hog1p (rabbit anti-Hog1p MAPK; Santa Cruz Biotechnology, Inc., USA) at a 1 : 1000 dilution or rabbit anti-phospho-p38 MAPK (Cell Signaling Technology, Beverly, MA, USA) at a 1 : 50000 dilution and against Pgk1p (mouse monoclonal anti-PGK1; Molecular Probes) at a 1 : 5000 dilution. This was followed by a one-hour incubation at room temperature with secondary antibody Peroxidase-AffiniPure Goat AntiRabbit IgG (1 : 10000; Jackson ImmunoResearch) or Peroxidase-AffiniPure Goat AntiMouse IgG (1 : 10000; Jackson ImmunoResearch).

## 3. Results and Discussion

### 3.1. NH_4_^+^-Induced Cell Death during Yeast Aging in Water Depends on the Specific Auxotrophy-Complementing Amino Acid Deprived from the Starvation Medium

In *Saccharomyces cerevisiae* BY4742, NH_4_^+^ leads to chronological life span (CLS) shortening, particularly relevant in cells starved for the auxotrophy-complementing amino acids simultaneously. The effect of NH_4_^+^ has been observed both in cells aged in spent culture medium limited for the essential amino acids and in cells aged in water after a 24-hour incubation in amino-acid-deprived medium [25]. We now sought to evaluate how the absence of specific auxotrophy-complementing amino acids affects NH_4_^+^ toxicity during yeast CLS. For this purpose, cells were first grown to exponential phase in SC medium and then starved for each of the three essential amino acids of the BY4742 strain (leucine, lysine, and histidine) alone or in combinations of two, as well as in their absence (aa-starved cells). As a control, we used the same medium, but without amino acid deprivation, therefore adding the three auxotrophy-complementing amino acids (nonstarved cells). Cells were then transferred to water with and without NH_4_^+^, and cell viability was evaluated over time. The protocol followed is systematized in Figure S1 in Supplementary material available online at http://dx.doi.org/10.1155/2013/161986.

**Figure 1:**
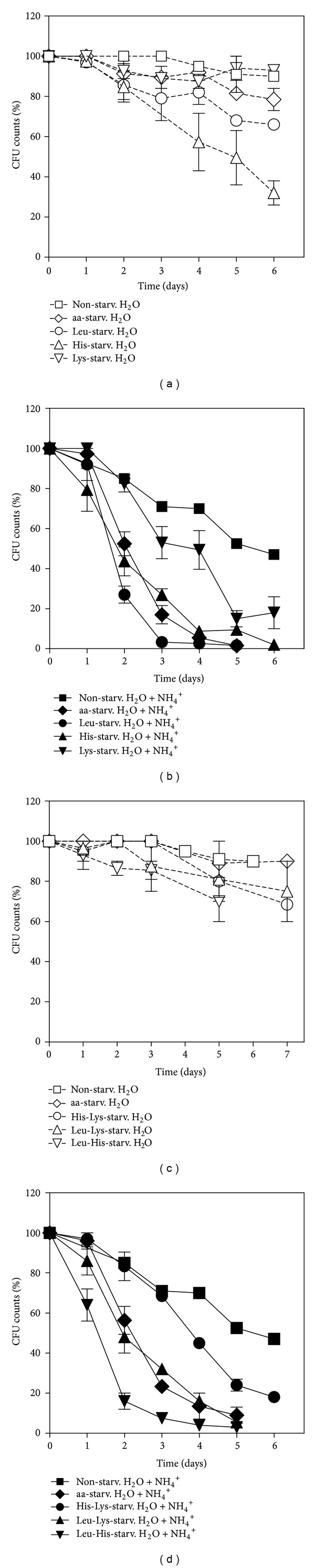
Ammonium-induced cell death during yeast aging in water is dependent on the specific auxotrophy-complementing amino acid deprived from the starvation medium. Survival of wild-type *S. cerevisiae* (BY4742) cells, nonstarved or starved for leucine, histidine, or lysine, in different combinations upon ((a) and (c)) transfer to water (open symbol) or ((b) and (d)) water with (NH_4_)_2_SO_4_, 0.5% (dark symbol). In all the cultures, starting cell density was about 3.8 × 10^7^ cells/mL, and the initial pH was adjusted to 7.0. Values are means ± SEM (*n* = 3). (b) **P < 0.01 (aa-starved H_2_O + NH_4_^+^ versus Lys-starved H_2_O + NH_4_^+^), **P < 0.01 (aa-starved H_2_O + NH_4_^+^ versus Leu-starved H_2_O + NH_4_^+^), and ***P < 0.001 (nonstarved H_2_O + NH_4_^+^ versus Lys-starved H_2_O + NH_4_^+^); (d) *P < 0.01 (nonstarved H_2_O + NH_4_^+^  versus His-Lys-starved H_2_O + NH_4_^+^), **P < 0.01 (aa-starved H_2_O + NH_4_^+^versus Leu-His-starved H_2_O + NH_4_^+^), and ***P < 0.001 (aa-starved H_2_O + NH_4_^+^ versus His-Lys-starved H_2_O + NH_4_^+^). Statistical analysis was performed by two-way ANOVA. All time points have error bars; however, for time points with reduced standard error, they are not visible.

The results presented in Figure 1(a) revealed that aa-, lysine- (Lys-), or nonstarved cells displayed a longer CLS in water without NH_4_^+^ than leucine- (Leu-) or histidine-(His-) starved cells. Furthermore, absence of any of the three amino acids in the medium, individually or at the same time, decreased CLS upon transfer of cells to water with NH_4_^+^, in comparison with the CLS of cells incubaed without amino acid restriction, though this effect was much less accentuated when only lysine was removed (Figure 1(b)). Two of the amino acids were then removed at the same time in different combinations (Figures 1(c) and 1(d)). Simultaneous absence of lysine and histidine (Lys-His-starved cells) had the least effect on NH_4_^+^-induced CLS shortening (Figure 1(d)), whereas NH_4_^+^ was most toxic to cells starved both for leucine and histidine (Leu-His starved cells). Comparing these results with those from Figure 1(b) (removal of one amino acid at a time from the medium), it can be observed that survival of Leu- or His-starved cells in water with NH_4_^+^ was much lower than that of cells that were also starved for lysine (Leu-Lys- or His-Lys-starved cells). On the other hand, the opposite effect was observed if Lys- or His-starved cells were simultaneously starved for leucine (Lys-Leu- or His-Leu-starved cells), where NH_4_^+^-induced CLS shortening was more severe. Additionally, histidine starvation in combination with one of the other two amino acids does not appear to have a major role in regulating CLS in response to NH_4_^+^, since the cell death profiles under those conditions were similar to those exhibited by Lys- or Leu-starved cells. 

Taken together, the results suggest that from the three auxotrophy-complementing amino acids tested, starvation for leucine alone or in combination with histidine resulted in the most severe effects on NH_4_^+^-induced CLS shortening, while starvation for lysine, alone or in combination with histidine, resulted in the less sensitive NH_4_^+^ phenotype.

### 3.2. Ras2p, Tor1p, and Sch9p Differently Mediate NH_4_^+^-Induced Cell Death during Yeast Aging in Water

The toxic effects of NH_4_^+^ in aa-starved BY4742 cells are the result of activation of the PKA and TOR pathways and are negatively regulated by Sch9p [25]. In addition, the results shown in the previous section demonstrated that ammonium affects CLS shortening depending on the specific essential amino acid deprived from the medium. We therefore sought to elucidate the role of Ras2/PKA, Tor1p, and Sch9p signaling pathways in CLS shortening induced by NH_4_^+^ under the different starvation conditions. For this, we first tested the effect of starving *tor*1Δ, *ras*2Δ, and *sch*9Δ cells for each of the three auxotrophy-complementing amino acids individually. As a control, we used the same medium in the absence or presence of all three essential amino acids. Similarly to what we described above, cells were first grown to exponential phase in SC medium, then incubated in the different starvation media, and next transferred to water with or without NH_4_^+^ (For schematic representation of the protocol please see Figure S1).

**Figure 2:**
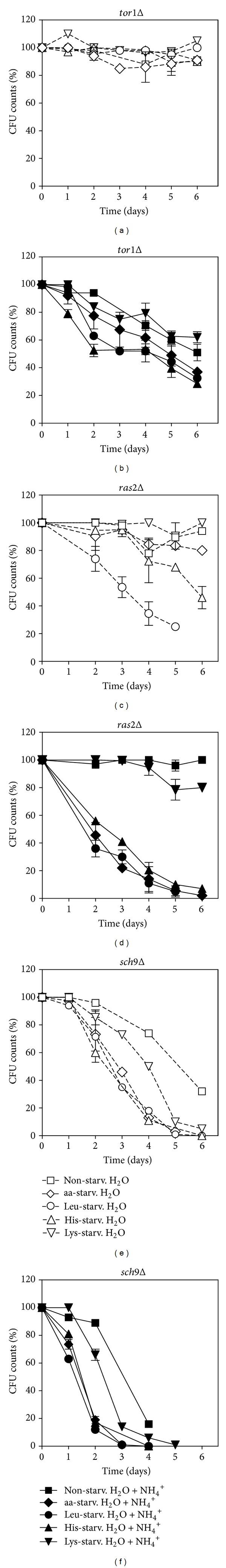
Tor1p regulates ammonium CLS shortening in response to amino acid starvation. Survival of ((a) and (b)) *tor1*Δ, ((c) and (d)) *ras2*Δ, and ((e) and (f))* sch9*Δ cells, nonstarved or starved for leucine, histidine, or lysine, individually or all at the same time, upon transfer to water (open symbol) or water with (NH_4_)_2_SO_4_, 0.5% (dark symbols). In all the cultures, starting cell density was about 3.8 × 10^7^ cells/mL, and the initial pH was adjusted to 7.0. Values are means ± SEM (*n* = 3). All time points have error bars; however, for time points with reduced standard error, they are not visible.

The *tor*1Δ strain displayed a lower NH_4_^+^-induced cell death than the wild-type strain in all starvation conditions tested (Figures 1(b) and 2(b)). Furthermore, Lys-starved cells displayed almost the same loss of cell viability as nonstarved cells in the presence of NH_4_^+^, showing that starvation for this amino acid does not induce sensitivity to NH_4_^+^ in the absence of *TOR1*. For nonstarved cells, there was no difference in the effect of NH_4_^+^ between wild-type and *tor*1Δ strains. On the other hand, deletion of *TOR1* also rescued the CLS of His-starved cells in water without NH_4_^+^ (Figures 1(a) and 2(a)).

In the *ras*2Δ strain, the loss of cell viability induced by NH_4_^+^ in nonstarved cells or Lys-starved cells was significantly reduced when compared with the wild-type strain. In contrast, deletion of *RAS2* had only a slight effect on the sensitivity of His- or Leu-starved cells to NH_4_^+^, as well as of cells starved for all three amino acids (aa-starved cells). Furthermore, for the last two starvation conditions, deletion of *RAS2* induced a strong shortening of CLS in water without NH_4_^+^, indicating that Ras2p is important to ensure longevity under these conditions (Figures 1(a) and 2(c)).

Absence of Sch9p reduced survival after cells were transferred to water with or without NH_4_^+^ for all conditions tested (starved or nonstarved). Data from Figures 2(e) and 2(f) show that Leu- or His-starved cells of the *sch*9Δ strain behaved as aa-starved cells when transferred to water with or without NH_4_^+^. In non- or Lys-starved cells, the loss of cell viability in water, with or without NH_4_^+^, was much less pronounced than in the other starvation conditions, as observed for wild-type cells.

### 3.3. Ras2p, Tor1p, and Sch9p Mediate PKA Activation in Response to NH_4_^+^ during Yeast Aging in Water

**Figure 3:**
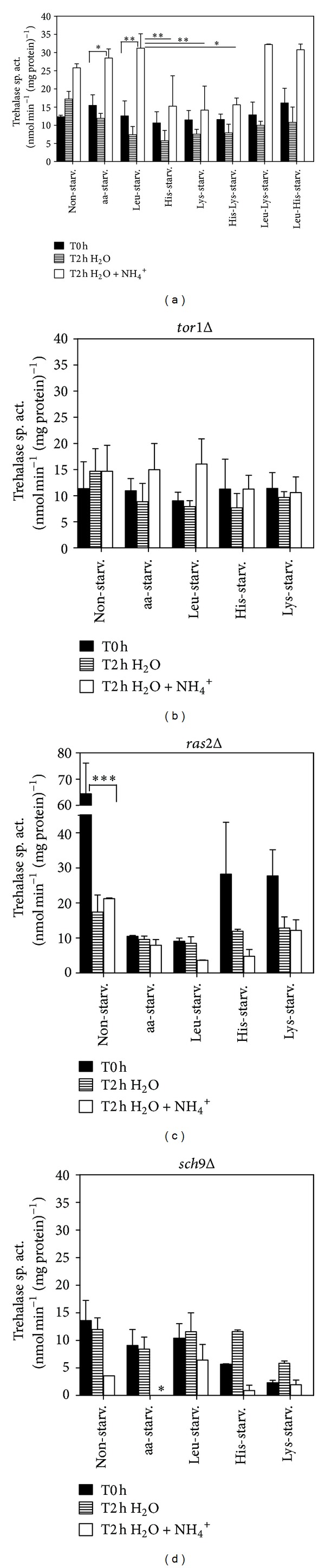
Ammonium induction of PKA activity depends on Tor1p, Ras2p and Sch9p regulation. Trehalase activity of cells nonstarved or starved for leucine, histidine, or lysine, individually or in different combinations, of (a) wild-type *S. cerevisiae* (BY4742) and of mutant deleted strains (b) *tor1*Δ, (c)* ras2*Δ, and (d)* sch9*Δ; before being, transferred to water (T0h) and after 2 hours in water (T2h H_2_O) or water with (NH_4_)_2_SO_4_, 0.5% (T2h H_2_O + NH_4_^+^). In all the cultures, starting cell density was about 3.8 × 10^7^ cells/mL, and the initial pH was adjusted to 7.0. Values are means ± SEM (*n* = 3–4). (a) *P < 0.05 (aa-starved T0h versus aa-starved T2h H_2_O + NH_4_^+^), **P < 0.01 (Leu-starved T0h versus Leu-starved T2h H_2_O + NH_4_^+^), **P < 0.01 (Leu-starved T2h H_2_O + NH_4_^+^ versus His-starved T2h H_2_O + NH_4_^+^), **P < 0.01 (Leu-starved T2h H_2_O + NH_4_^+^ versus Lys-starved T2h H_2_O + NH_4_^+^), *P < 0.05 (Leu-starved T2h H_2_O + NH_4_^+^ versus His-Lys-starved T2h H_2_O + NH_4_^+^); (c) ***P < 0.001 (nonstarved T0h versus nonstarved H_2_O + NH_4_^+^); (d) *P < 0.05 (aa-starved T0h versus aa-starved T2h H_2_O + NH_4_^+^). Statistical analysis was performed by two-way and one-way ANOVA.

To further evaluate the role of PKA in NH_4_^+^-induced CLS shortening and the potential effects of Tor1p, Ras2p, and Sch9p as PKA upstream regulators, we assessed PKA activation in BY4742 (wild-type), *tor*1Δ, *ras*2Δ, and *sch*9Δ strains starved for each of the three essential amino acids, individually or in combination. Trehalase is a target of PKA regulation, and its activity has been extensively used to monitor PKA activation [22, 29]. In order to evaluate PKA activation, we therefore measured trehalase activity in cells grown and incubated as described above in material and methods section. (For schematic representation of the protocol please see Figure S1). We observed that in wild-type cells, leucine starvation resulted in the highest trehalase activity after 2 h of incubation with NH_4_^+^, whereas its presence alone led to the lowest trehalase activity. In contrast, and under the same conditions, starvation for lysine or histidine alone gave rise to the lowest trehalase activity, whereas their presence alone led to the highest trehalase activity (Figure 3(a)). The results also showed that in the presence of NH_4_^+^, aa-starved cells exhibited a PKA activation pattern similar to nonstarved cells, with values that are between those obtained for Leu- and His- or Lys-starved cells. This suggests that in aa-starved cells, the higher contribution expected from PKA activation due to the absence of leucine is probably balanced by the decrease of PKA activity induced by the absence of histidine and lysine. PKA activation by NH_4_^+^ was decreased in *tor*1Δ, *ras*2Δ, and *sch*9Δ mutants in comparison with the wild-type strain, both for nonstarved cells and under all amino acid starvation conditions (Figures 3(b), 3(c), and 3(d)). The observed reduction in PKA activation correlates with the decrease in NH_4_^+^-induced CLS shortening in the *tor*1Δ strain under all the conditions tested. In addition, the decrease in PKA activation induced by NH_4_^+^ in the *ras2*Δ strain was accompanied by an increase in cell survival for non- or lysine-starved cells, but not for cells under the remaining starvation conditions (Leu-, His-, or aa-starved cells). Conversely, for nonstarved cells and for cells starved in the presence of leucine (His-starved and Lys-starved cells) before transfer to water (T0), there was a significant increase in PKA activation in the *ras*2Δ strain, indicating that Ras2p seems to downregulate PKA activity in the presence of leucine. On the other hand, the decrease in PKA activation induced by NH_4_^+^ in the *sch9*Δ strain was not associated with an extended CLS in water with NH_4_^+^ in nonstarved cells or under any of the starvation conditions tested, which is in accordance with previous results described for aa-starved cells [25]. Together, the results suggest that NH_4_^+^ induces PKA activation through Tor1p, Ras2p, and Sch9p. However, absence of Ras2p, although able to decrease PKA activation, did not revert NH_4_^+^-induced CLS shortening in Leu-, His-, and aa-starved cells, indicating that in the absence of this protein, other pathways, independent of PKA and possibly mediated by Tor1p, are still activated and can induce cell death. Furthermore, Ras2p, at least under some conditions, appears to also activate other pathways relevant to cell survival, since its absence leads to a shorter CLS in water. A prosurvival role was also observed for Sch9p under all conditions, either in the absence or presence of NH_4_^+^.

### 3.4. Sch9p Protects Cells from NH_4_^+^-Induced Cell Death through Hog1p Activation

**Figure 4:**
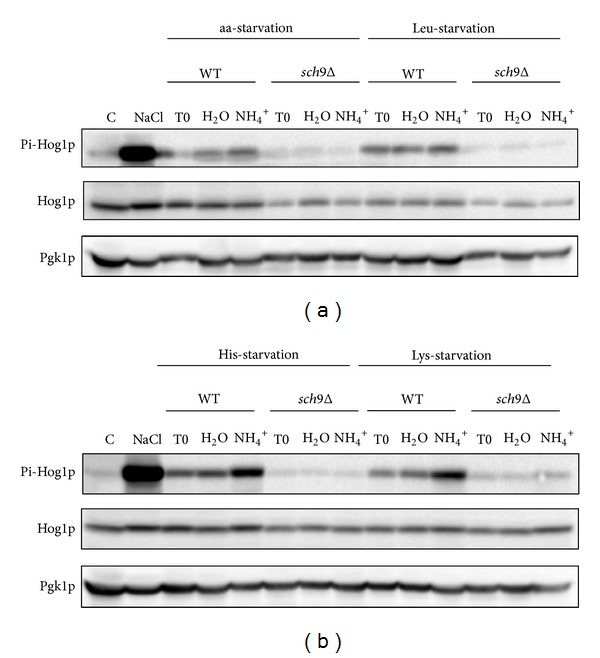
Ammonium induces Sch9p-dependent Hog1p phosphorylation in starvation conditions. Westernblot analysis of Pi-Hog1p levels present in *S. cerevisiae *(BY4742) wild-type (WT) and* sch9*Δ cells starved for (a) all three amino acids or leucine and (b) starved for histidine or lysine, before (T0) and after 20 minutes upon transfer to water (H_2_O) or water with (NH_4_)_2_SO_4_, 0.5% (NH_4_^+^). In all the cultures, starting cell density was about 3.8 × 10^7^ cells/mL, and the initial pH was adjusted to 7.0. Control cells were grown on YPD medium (Control-C) and incubated for 5 minutes in YPD medium supplemented with 1 M NaCl.

Hog1p is a kinase that regulates and is regulated by Sch9p and mediates stress response independently of the PKA and TOR pathways [30]. It was previously shown that Hog1p is involved in the resistance of aa-starved cells to the toxic effects of NH_4_^+^ during CLS in water [25]. In order to access if the protective role of Sch9p in response to NH_4_^+^ under the different amino acid starvation conditions described in the previous sections could be mediated through a Sch9p-dependent Hog1p activation, we examined Hog1p phosphorylation during CLS in water with and without NH_4_^+^ in BY4742 (wild-type) and *sch*9Δ cells. As shown in Figure 4, Hog1p phosphorylation in wild-type cells increased in the presence of NH_4_^+^ in all starvation conditions tested (aa-, Leu-, His-, and Lys-starved cells), being higher in His- and Lys-starved cells. Deletion of *SCH9* almost abolished Hog1p phosphorylation in aa-, Leu-, and His-starved cells, whereas some residual phosphorylation was still detected in Lys-starved cells. The lower Hop1p phosphorylation observed for cells starved for aa- and Leu-starved cells is in agreement with previous results, showing that the presence of leucine is required for Sch9p phosphorylation via TORC1 [21]. Also, Hog1p phosphorylation in Lys-starved *sch*9Δ cells is in agreement with the activation of pathways other than the PKA in the absence of Ras2p, suggested by the rescue of the loss of viability found for Lys-starved *ras*2Δ cells (Figure 2(d)).

Taken together, results show that Sch9p is involved in Hog1p activation in response to NH_4_^+^ under all starvation conditions, indicating that the increased resistance afforded by Sch9p could, actually, be mediated through Hog1p activation.

## 4. Conclusions

It has been previously shown that the CLS of stationary phase cells of *Saccharomyces cerevisiae* BY4742 transferred to water was significantly shortened by the addition of NH_4_^+^ and that the negative effects of NH_4_^+^ were particularly evident for cells under restriction of auxotrophy-complementing amino acid markers (leucine, lysine, and histidine) [25]. The results presented herewith demonstrate that NH_4_^+^-induced cell death during aging in water depends on the specific auxotrophy-complementing amino acid deprived from the starvation medium. While Lys-starved cells were only slightly more sensitive to NH_4_^+^-induced CLS shortening than nonstarved cells, Leu- and His-starved cells displayed a much stronger sensitivity to NH_4_^+^ during CLS, which was comparable to that previously described for cells simultaneously starved for all three essential amino acids (aa-starved cells). When we compare cells starved for one amino acid at a time with nonstarved cells, absence of any of the three auxotrophy-complementing amino acids from the medium has a detrimental effect leading to a faster loss of cell survival in response to ammonium. However, when cells are starved for at least one amino acid, the presence of lysine in the medium is detrimental, histidine does not seem to have an effect, and leucine has a protective effect on ammonium-induced CLS shortening. The results regarding leucine are in accordance with the literature since it has been described that leucine plays a more important role in CLS extension in auxotrophic strains [6, 10]. In a recent study, supplementation of extra leucine to SC medium or transformation of auxotrophic leucine strain into a prototrophic leucine strain resulted in CLS extension. The importance of leucine was attributed to the regulation of the branched side chain amino acids synthesis that appears to be misregulated in a *leu2*Δ strain. In agreement, supplemental levels of the branch side amino acids isoleucine, threonine, and valine also extended CLS in a *leu2*Δ strain [6]. The negative effect observed for lysine in cell survival during ammonium-induced cell death can possibly be due to the fact that autophagy is inhibited in the presence of ammonium [25], and the lack of autophagy might be responsible for this effect since lysine seems to act in an autophagy-dependent manner on the regulation of CLS. Autophagy-deficient strains showed no improvement in CLS extension after regaining LYS prototrophy in contrast to wild-type autophagy competent cells that increased CLS extension with LYS prototrophy [6]. 

Both Ras2p and Tor1p are involved in NH_4_^+^-induced CLS shortening in aa-starved cells [25]. We now further established that Ras2p involvement on NH_4_^+^-induced CLS shortening was present under all conditions tested, and did not depend on starvation. In turn, Tor1p function in the decrease of CLS by NH_4_^+^ was relevant only under amino acid starvation, being differently modulated by the specific amino acid deprived from the medium. Starvation for leucine and histidine, which induced a strong shortening of CLS in the presence of NH_4_^+^, had a high impact in the regulation of Tor1p function, whereas starvation for lysine, which was associated with only a small NH_4_^+^-induced CLS shortening, had a considerably less significant impact on Tor1p regulation. These results are in agreement with previous results showing that leucine has an important impact in the regulation of TORC1 [20, 21]. Our results suggest that the presence of NH_4_^+^ in the medium (commonly present as the nitrogen source) may be at least partly responsible for the reported decrease in CLS in leucine-starved cells [6, 14].

PKA activation has been described to be associated with the NH_4_^+^-induced CLS shortening of aa-starved cells in water [25]. From the results now obtained, and when we compare values from nonstarved and starved wild-type cells, it appears that leucine starvation (alone or in combination with starvation for another amino acid) is the main factor responsible for PKA activation in response to NH_4_^+^, correlating with its stronger effect on CLS shortening. This activation is dependent on Ras2p, Tor1p, and Sch9p, as deficiency in any of these proteins leads to its decrease. However, since the decrease in PKA activation resulted in distinct cell fate outcomes in the different mutants, the results suggest that these proteins activate PKA by independent pathways and/or also regulate other pathways that they do not share and that have different impacts on NH_4_^+^-induced CLS shortening. Also, we cannot exclude the possibility that the observed effects on trehalase activity may result from a potential effect of Sch9p, Ras2p, or Tor1p on the activity of other proteins also involved in trehalase regulation such as Bmh1/2p or Dcs1p [29].

Opposite to our results, Sch9p has been described to inhibit PKA activity when glucose is added to glycerol-grown cells. However, these authors observed that the inhibition was mediated through the regulation of Tpk2p localization [31], an isoform that does not seem to have a relevant role in response to ammonium under our conditions. In fact, we have previously observed that Tpk1p is the main PKA isoform involved in ammonium effects [25]. In addition to its involvement in PKA activation, Sch9p also increases Hog1p phosphorylation, extending CLS in water with or without NH_4_^+^. 

In summary, herewith we show that the toxic effects of NH_4_^+^ on CLS shortening are regulated by a starvation-dependent and a starvation-independent component and are mediated essentially by Tor1p in the first case and by Ras2p in the second. We also provide evidence that when cells are starved for amino acids, the presence of leucine can ameliorate NH_4_^+^-induced CLS shortening, while lysine has the opposite effect, and the presence of histidine has no effect. Together, our data add new knowledge on CLS regulation, indicating that the modulation of nitrogen sources supplied to cells can drastically modulate CLS and providing new clues for the development of environmental interventions for chronological life span extension. Additionally, and since NH_4_^+^-induced cell death is involved in different human disorders that are accompanied by hyperammonemia [32], our results, showing that NH_4_^+^ toxicity can be modulated by amino acids through different pathways, may also afford new insights into the understanding of the cell molecular bases triggering cell death in such pathologies.

## Supplementary Material

Schematic representation of the protocol used for the different starvation conditions tested.
