# Supplementary material for: Ammonium-Dependent Shortening of CLS in Yeast Cells Starved for Essential Amino Acids Is Determined by the Specific Amino Acid Deprived, through Different Signaling Pathways
Source: Oxid Med Cell Longev. 2013 Aug 26;2013:161986. doi: 10.1155/2013/161986 (PMC3767051; doi:10.1155/2013/161986)
Supplement: Supplementary file 1 — Schematic representation of the protocol used for the different starvation conditions tested. [file 161986.f1.pdf]

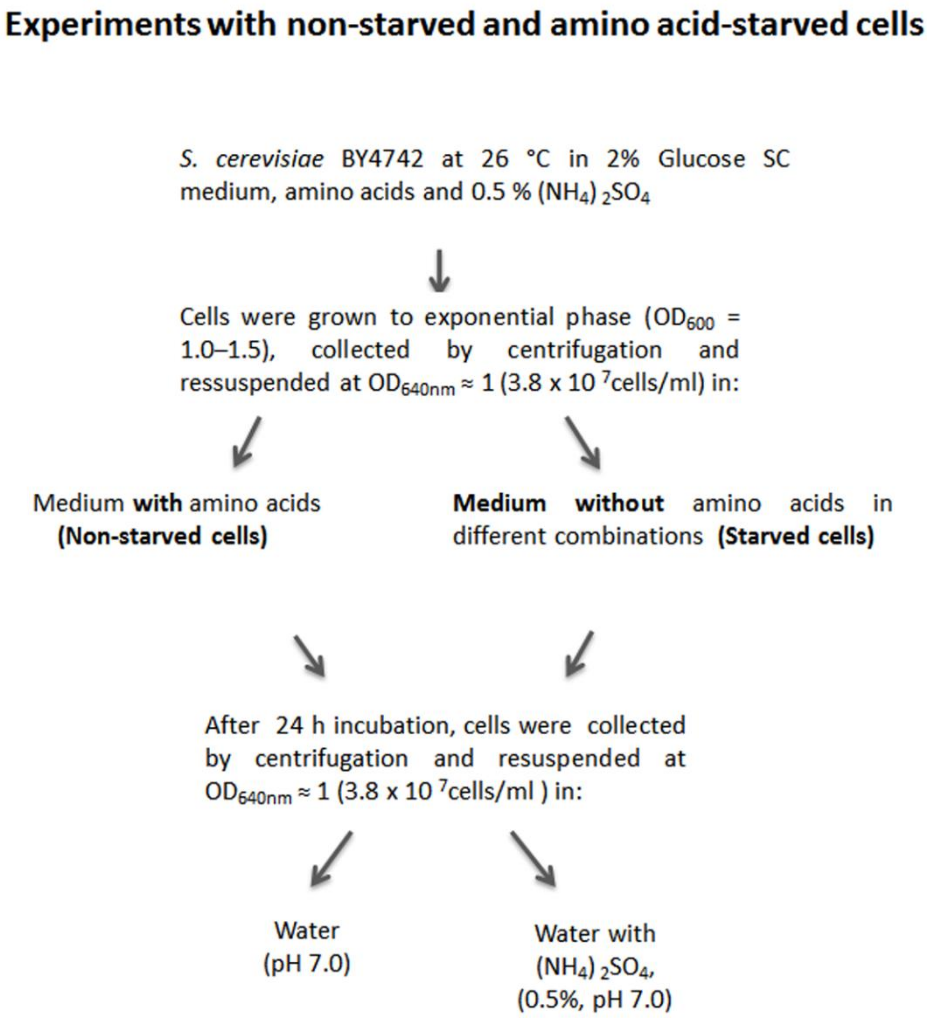

Figure S1. Scheme of the methodology used in experiments with Non-starved and starved cells.
